# Supplementary material for: Fast and efficient QTL mapper for thousands of molecular phenotypes
Source: Bioinformatics. 2015 Dec 26;32(10):1479–85. doi: 10.1093/bioinformatics/btv722 (PMC4866519; doi:10.1093/bioinformatics/btv722)
Supplement: Supplementary Data [file supp_32_10_1479__index.html]

Fast and efficient QTL mapper for thousands of molecular phenotypes — Fast and efficient QTL mapper for thousands of molecular phenotypes — Fast and efficient QTL mapper for thousands of molecular phenotypes — Supplementary Data 

# Fast and efficient QTL mapper for thousands of molecular phenotypes

## Supplementary Data

files

- Supplementary Data - doc file
- Supplementary Data - doc file
