# Supplementary material for: Fast and efficient QTL mapper for thousands of molecular phenotypes
Source: Bioinformatics. 2015 Dec 26;32(10):1479–85. doi: 10.1093/bioinformatics/btv722 (PMC4866519; doi:10.1093/bioinformatics/btv722)
Supplement: Supplementary Data [file supp_btv722_fastQTL_B_suppFigures.doc]

**Supplementary figures**

**for**

***Fast and efficient QTL mapper for molecular phenotypes***

**by H. Ongen et al.**

**1. Supplementary figure 1**


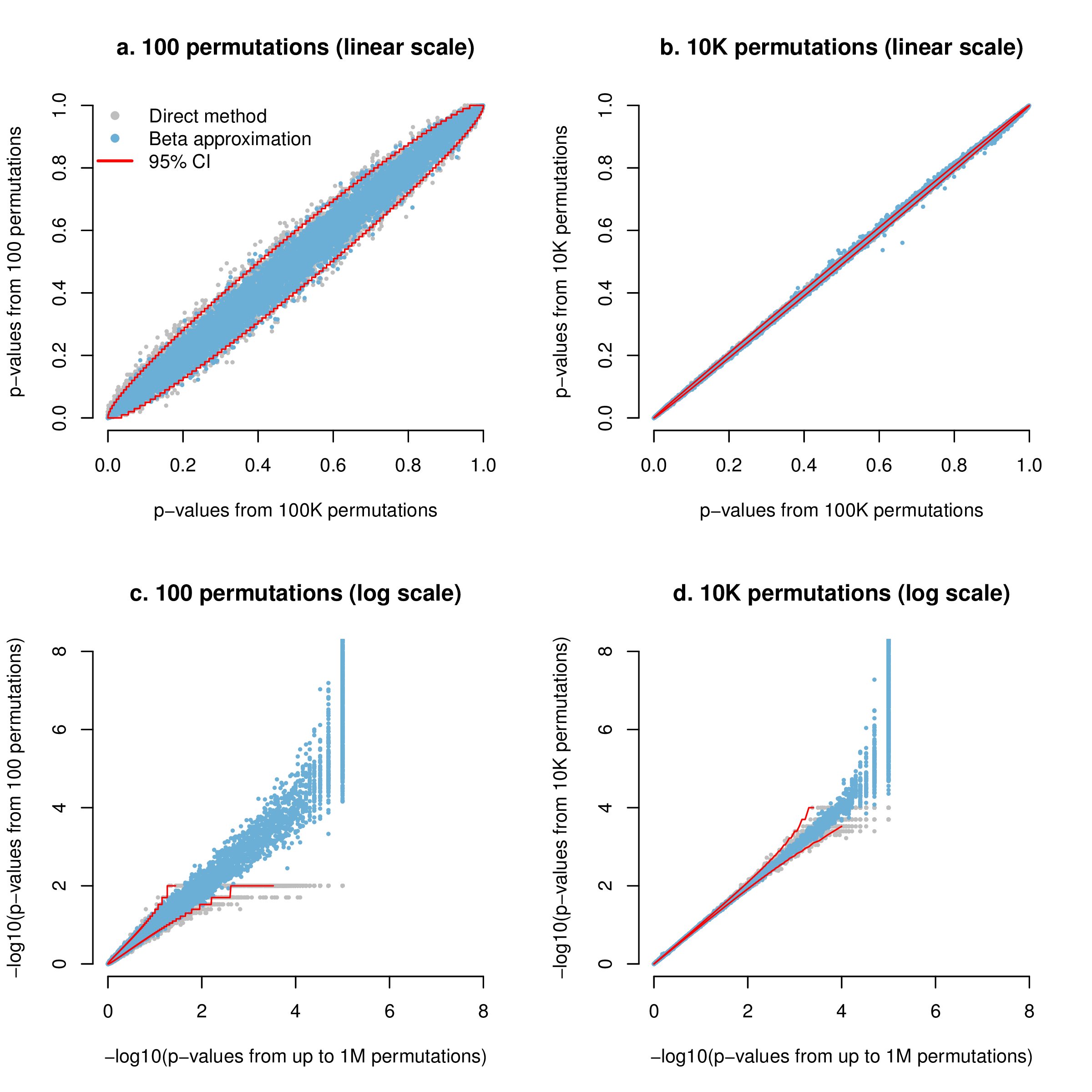
Calibration of the Beta approximated p-values varying the number of permutations: Scatter plot of adjusted p-values obtained with 100 and 10K permutations (x-axis; the 1,000 permutations case is shown in main figure 3a-b) via the direct method (in grey) and the beta approximation (in light blue) against those obtained through standard method from 100K permutations (y-axis) for panels (a, b) and from 1K to 1M for panel (c, d). Adjusted p-values are plotted on a normal scale (a, b) to focus on the most insignificant corrected p-values and on a log scale (c, d) to focus on the most significant corrected p-values. Note that y-axis in panels (b, d) have been truncated to only show points between 0 and 8. Expected sampling variation for the corresponding number of permutations is characterized by 95% confidence intervals shown in red.

**2. Supplementary figure 2**


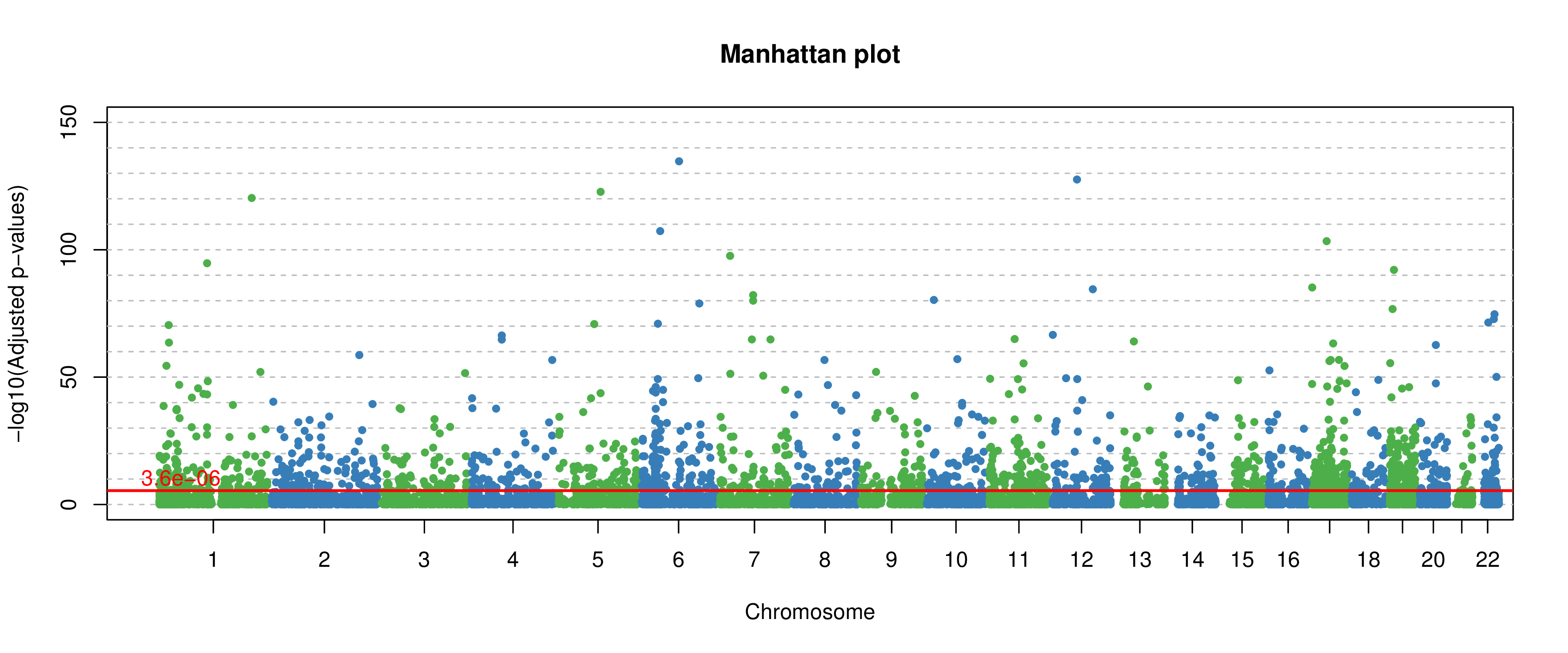
Genome-wide distribution of Beta approximated p-values: Manhattan plot of the adjusted p-values obtained by Beta approximation from 1K permutations on GEUV_EUR. Each point corresponds to a gene (13,703 genes in total) with the -log10 of the corrected p-value of the candidate QTL shown (y-axis) as a function of the genomic location (x-axis). The Bonferroni threshold of significance is shown with a solid red line.

**3. Supplementary table 1**

Data summary: This table gives, for each data set, the ID used throughout the paper, the consortium the data belongs to, the ancestry of the samples, the cell or tissue type in which RNA-seq has been performed, the number of samples, the number of quantified genes passing QC and the number of variants passing QC.
