# Supplementary material for: Fast and efficient QTL mapper for thousands of molecular phenotypes
Source: Bioinformatics. 2015 Dec 26;32(10):1479–85. doi: 10.1093/bioinformatics/btv722 (PMC4866519; doi:10.1093/bioinformatics/btv722)
Supplement: Supplementary Data [file supp_btv722_fastQTL_B_suppMaterials.doc]

**Supplementary materials**

**for**

***Fast and efficient QTL mapper for molecular phenotypes***

**by H. Ongen et al.**

1. The Geuvadis data set

The Geuvadis data in our experiments corresponds exactly to what has been used to map eQTLs in the original study, so please consult the supplementary materials of the corresponding paper for a more detailed description of the data preparation steps (Lappalainen et al. 2013). The Geuvadis data contains two distinct populations: one comprising 89 Yorubans and the other 373 Europeans (GEUV_EUR): we only used the second one in this paper. As molecular phenotypes, gene expression levels quantified using RNA-seq were used. Poor gene quantifications were filtered out (all genes quantified for less than 90% of the samples); the remaining ones were normalized with PEER (Stegle et al. 2012) for 10 confounding factors and quantile-normalized to be normally distributed using the *R/rntransform* function of the GenABEL package (Aulchenko et al. 2007). As a results, a total of 13,703 genes were available for analysis. Genotype data was obtained via low coverage sequencing by the 1,000 Genomes project consortium (1000 Genomes Project Consortium. 2012) for most of the samples and via imputation into Illumina OMNI2.5M for the rest. Variant sites with a minor allele frequency below 5% were filtered out, resulting in a set of 10,785,346 variant sites genome-wide containing a mixture of SNPs, short bi-allelic indels and large deletions. A summary of this data set is shown in supplementary table 1.

**2. The GTEx pilot data set**

The GTEx data we used in our experiments corresponds exactly to what has been used to map eQTLs in the pilot study, so please consult the supplementary materials of the corresponding paper for a more detailed description (GTEx Consortium. 2015). The GTEx pilot compiles data for nine tissue types; adipose (subcutaneous), tibial artery, heart (left ventricle), lung, muscle (skeletal), tibial nerve, skin (sun exposed), thyroid, and whole blood. The sample size per tissue ranges from 83 to 156. As molecular phenotypes, gene expression levels were quantified using RNA-seq. Poor gene quantifications were filtered out (RPKM>0.1 in 10 samples at least is required); remaining ones were quantile-normalized to be normally distributed. In total, 28,992 to 34,884 genes were considered in the analysis, depending on the tissue. All samples were genotyped using Illumina Omni 5M followed by imputation from 1000 Genomes phase I. After standard QC (ex: MAF > 5%), a total of 6,820,472 variant sites were used in the analysis. A summary of this data is shown in supplementary table 1. Discovery of eQTLs in GTEx pilot was done within +/- 1Mb of the gene TSS using linear regressions as implemented in Matrix eQTL with as covariates (a) the first three principal components to account for population stratification in the genotype data and (b) 15 PEER factors to account for batch effects in the expression data. To control for multiple testing, gene quantification were permuted between 1,000 and 10,000 times depending on the significance of the gene (adaptive permutation scheme with B=15) and FDR controlled at 5% using the ST procedure as implemented in R/qvalue. In total, between 948 and 2,370 eQTLs were discovered depending on the tissue type.

**3. Equivalent number of permutations**

In this section, we describe the method used to estimate the *equivalent* number of permutations shown in figure 3c, which is the number of permutations that the direct permutation scheme needs to reach the same level of accuracy as the beta approximated p-values. In this approach, we consider as true *adjusted* p-values those obtained from the direct permutation scheme using 1,000,000 permutations. Specifically, we only consider p-values in the range 10^-5^ to 1.0 to only keep those that are well estimated from 1,000,000 permutations. Then, we sort and bin all the p-values in this range such that each bin contains 200 p-values. For each bin, we calculate the mean p-value *μ* as the x-axis coordinate and find by exhaustive search the smallest and largest number of permutations providing a 95% confidence interval that contains 95% of the Beta approximated p-values. We report as y-axis coordinate the midpoint of the interval found. In other words, we explore various numbers of permutations to find the corresponding 95% confidence interval which contains 95% of the beta approximated p-values. In practice, we use the following functions in R to estimate the lower and upper bound of the 95% confidence interval resulting from *R* permutations: qbinom(0.025, R, μ) / R and qbinom(0.975, R, μ) / R.

**4. Running Matrix eQTL**

Here, we describe how to efficiently run Matrix eQTL (Shabalin. 2012) on the Geuvadis & GTEx data sets we used in this work. To this end, we recompiled R v3.1.2 (<http://cran.r-project.org/src/base/R-3/R-3.1.2.tar.gz>) such that it includes the ACML v5.3.1 library (<http://developer.amd.com/tools-and-sdks/cpu-development/amd-core-math-library-acml/>), a version of the BLAS library optimized for AMD CPUs. Linking R to the ACML library is crucial to get good performance of Matrix eQTL since it usually provides in our experiments a ~10 fold speed up (data not shown) compared to the default BLAS library provided with R. Then, we split all 9 phenotype and genotype data sets by chromosome (i.e. 22 chunks per data set) in order to increase the IO efficiency and the parallelization granularity of Matrix eQTL while keeping enough phenotypes to be processed simultaneously to leverage the matrix design of Matrix eQTL. Next, we developed an R script embedding Matrix eQTL v2.1.0 and implementing the *direct* permutation scheme as follows: (1) the phenotype/genotype data for a chromosome are read just once and loaded in RAM, (2) a nominal pass of association is performed for all variant-phenotype pairs within a *cis*-window of 1Mb and using the same covariates as in the FastQTL runs and (3) a user-defined number of permutation passes is performed. Finally, a single *adjusted* p-value per molecular phenotype is produced using equation (1). Note that each Matrix eQTL run (nominal and permutation passes) uses the following set of options [*useModel=modelLINEAR, pvOutputThreshold_cis=1e-6, pvOutputThreshold=0, cisDist=1e6, noFDRsaveMemory=TRUE, min.pv.by.genesnp=TRUE*]*.*

**References**

1000 Genomes Project Consortium, Abecasis GR, Auton A, Brooks LD, DePristo MA, Durbin RM, Handsaker RE, Kang HM, Marth GT, McVean GA. 2012. An integrated map of genetic variation from 1,092 human genomes. Nature. 491:56-65.

Aulchenko YS, Ripke S, Isaacs A, van Duijn CM. 2007. GenABEL: an R library for genome-wide association analysis. Bioinformatics. 23:1294-6.

GTEx Consortium. 2015. The Genotype-Tissue Expression (GTEx) pilot analysis: multitissue gene regulation in humans. Science. 348:648-60.

Lappalainen T, Sammeth M, Friedländer MR, 't Hoen PA, Monlong J, Rivas MA, Gonzàlez-Porta M, Kurbatova N, Griebel T, Ferreira PG, et al. 2013. Transcriptome and genome sequencing uncovers functional variation in humans. Nature. 501:506-11.

Shabalin AA. 2012. Matrix eQTL: ultra fast eQTL analysis via large matrix operations. Bioinformatics. 28:1353-8.

Stegle O, Parts L, Piipari M, Winn J, Durbin R. 2012. Using probabilistic estimation of expression residuals (PEER) to obtain increased power and interpretability of gene expression analyses. Nat Protoc. 7:500-7.
